# Symptomatic presentation with cervical cancer in Uganda: a qualitative study assessing the pathways to diagnosis in a low-income country

**DOI:** 10.1186/s12905-015-0167-4

**Published:** 2015-02-18

**Authors:** Amos Deogratius Mwaka, Elialilia Sarikiaeli Okello, Henry Wabinga, Fiona M Walter

**Affiliations:** Department of Medicine, School of Medicine, College of Health Sciences, Makerere University, PO Box 7072, Kampala, Uganda; Department of Psychiatry, School of Medicine, College of Health Sciences, Makerere University, PO Box 7072, Kampala, Uganda; Kampala Cancer Registry, Department of Pathology, School of Biomedical Sciences, College of Health Sciences, Makerere University, Kampala, Uganda; The Primary Care Unit, Department of Public Health & Primary Care, University of Cambridge, Cambridge, UK

**Keywords:** Cervical cancer, Help-seeking, Illness attributions, Model of Pathways to Treatment

## Abstract

**Background:**

Symptomatic cervical cancer patients in low- and middle-income countries usually present with late stage disease and have poor survival. We explored the views of cervical cancer patients on their symptom appraisal and interpretations, and their help-seeking including lay consultations.

**Methods:**

We conducted an in-depth interview study in two northern Ugandan hospitals. Theoretical models underpinned the study guide for data collection and analysis. We used thematic analysis techniques, informed by the theoretical concepts in the Model of Pathways to Treatment. Sub-themes and themes were identified through consensus among investigators.

**Results:**

Eighteen women aged 35–56 years, recently diagnosed with cervical cancer were interviewed. Their first symptoms included abnormal vaginal bleeding, offensive vaginal discharge and lower abdominal pain. Most participants did not perceive themselves to be at risk for cervical cancer and they usually attributed the initial symptoms to normal bodily changes or common illnesses such as sexually transmitted diseases. Lay consultations with husbands, relatives and friends were common and often influenced decisions and timing for seeking care. Prompt help-seeking was frequently triggered by perceived life threatening symptoms such as heavy vaginal bleeding or lower abdominal pain; symptom burden sufficient to interfere with patients’ work routines; and persistence of symptoms in spite of home-based treatments. Participants did not promptly seek care when they perceived symptoms as mild; interpreted symptoms as due to normal bodily changes e.g. menopause; and attributed symptoms to common illnesses they could self-manage. Their cancer diagnosis was often further delayed by long help-seeking processes including repeated consultations. Some healthcare professionals at private clinics and lower level health facilities failed to recognize symptoms of cervical cancer promptly therefore delayed referring women to the tertiary hospitals for diagnosis and treatment.

**Conclusion:**

Ugandan patients with symptomatic cervical cancer often misattribute their gynaecological symptoms, and experience long appraisal and help-seeking intervals. These findings can inform targeted interventions including community awareness campaigns about cervical cancer symptoms, and promote prompt help-seeking in Uganda and other low- and middle-income countries with high incidence and mortality from cervical cancer.

## Background

In Uganda and most low- and middle-income countries (LMICs), there are no organized population-based cervical screening programs, largely due to financial and human resource restraints [1,2]. Consequently, cervical cancer patients in low- and middle–income countries (LMIC) report late for medical care [3-5], experience less treatment benefits and have poor survival [6,7]. Little is known about the reasons why cancer patients present to healthcare at a later stage in the LMICs than the high-income countries (HICs). In our recent Ugandan interview study, healthcare professionals suggested that advanced stage at diagnosis could be related to many challenges including patient factors such as inadequate awareness about cervical cancer symptoms, and healthcare factors such as inadequate skills to diagnose cervical cancer, inaccessibility of primary and secondary healthcare facilities, and a lack of specialized clinicians including pathologists and gynaecological oncologists [8].

Analysis of national survey data on common cancers in the UK showed that patient and primary care intervals (defined respectively as the time from the patient first noticing a symptom, to presenting to primary care, and the time from then to being referred to a specialist) were much longer than referral and secondary care intervals (the time from being referred to being diagnosed) [9]. In a qualitative study set in Australia, rural cancer patients delayed seeking healthcare because of long distances to health facilities [10]. These findings suggest a need to better understand the patient and primary care intervals in order to design interventions for prompt healthcare seeking and diagnosis. However, transferring findings from studies in the HICs to the LMICs needs be done with caution because of the contextual differences between the healthcare systems of the HICs and LMICs. And yet in Uganda and most sub-Saharan African countries, there are few data from qualitative studies assessing the help-seeking process for cervical cancer [11,12].

Qualitative studies, particularly when guided by theoretical models, can provide useful insights into patient views, and guide interventions on help-seeking for cancers and other conditions [13,14].The Andersen model has been a fairly widely used theoretical approach which posits that patients go through a number of stages, referred to as delays, when they experience persistent and or worsening bodily sensations or symptoms [15,16]. The Andersen model was recently reviewed for its application to cancer studies, and refined into the Model of Pathways to Treatment [16,17]. This model proposes that an individual’s route through symptom appraisal and help-seeking is a nonlinear, iterative process with definable events, intervals (appraisal, help-seeking, diagnostic, and pre-treatment) and processes [16,17].

This study aimed to explore the process of symptom appraisal and help-seeking for symptoms of cervical cancer in Uganda using the Model of Pathways to Treatment as a framework for analysis. Understanding the patient journey to presentation along the pathway to diagnosis and treatment of cervical cancer can allow the identification of the critical intervals and barriers within the pathway, elucidate the nature of the barriers, and inform targeted interventions to minimize such barriers and improve timely presentation and diagnosis of symptomatic cervical cancer.

## Methods

### Design

An interview-based, qualitative design was chosen because it provided us an opportunity to explore cervical cancer patients’ symptoms experiences, and gain detailed understanding about their symptom-appraisal and help-seeking [18,19].

### Setting

Patients attending care at a private-not-for-profit (PNFP) and a public Regional Referral Hospital, both in Gulu, northern Uganda.

### Recruitment

Cervical cancer patients diagnosed within three months and attending care in the gynaecology clinics and wards were recruited to take part in a survey to determine stage distribution and factors associated with advanced stage cervical cancer. Patients were approached by the research nurse assistant, after clinical diagnosis of cervical cancer and during in-patient admission for examination under anaesthesia (EUA) and histological confirmation. The nurse was trained in aspects of the study including objectives, recruitment and consent procedures, and basic facts about cervical cancer including prevention methods, risk factors, symptoms and treatment modalities. The date of the EUA for diagnosis and staging of cervical cancer provided the date of diagnosis. Among women identified via a survey as willing to be interviewed, we purposively sampled patients who were not too unwell (as determined by the research assistant in consultation with the attending gynaecologists) to participate in an interview and who spoke the local language. Other exclusion criteria included asymptomatic patients identified with suspected cervical cancer through screening, and patients who had disabilities making communication difficult such as hearing and speech problems or a clinical diagnosis of a mental illness. We recruited in two phases. The first phase lasted six weeks and started at the beginning of the survey in August 2012. Ten patients completed the survey during this time period, nine spoke the local language, and we interviewed six of these nine patients. The data collected from these first six patients were transcribed, translated and analysis started to identify emerging themes. We also sought to identify deviant or disconfirming cases to inform further data collection. The second phase of data collection was undertaken from May 2013 for 8 weeks. The same purposive sampling approach was used, and among the 24 patients who completed the survey during this time period, 23 were identified as speaking the local language, of whom12 agreed to be interviewed. As the preliminary analysis suggested that two participants who were aware of their HIV positive status attributed their initial symptoms differently and undertook a different help-seeking pathway from the other women, they were considered deviant cases. In the second phase one further HIV positive participant was interviewed; no new themes emerged, therefore we considered that we had achieved saturation of data and were able to stop recruitment at that point.

### Interview guide

An interview guide was used to aid data collection (Table 1). The development of the guide was informed by the constructs in the General Model of Total Patient Delay [20]. It included items on recognition and appraisal of symptoms, durations and causal attributions of symptoms, immediate actions taken to deal with symptoms, where care was sought and why, people consulted, triggers for help-seeking, intervals between various actions taken, and items on perceptions about cervical cancer causes, group at risk, personal perceived risk for cervical cancer, and perceptions of treatment practices and outcomes. The guide was developed in English then translated to Acholi, the local language widely spoken in the study area. Two people (a social scientist and an educationist) independently performed the translations. The two Acholi versions were discussed and merged into one document which was back translated into English to evaluate accuracy in translation and preserve conceptual equivalence. The guide was refined accordingly before translation back into Acholi.

**Table 1:** **In-depth interview guide**

**Thematic area**	**Guiding questions**	**Prompts**
**1. Disease labelling and symptoms; Appraisal stage**	(a) Please tell me about the illness you have which is now known to be cervical cancer.	(i) What were the first symptoms at onset of the illness?
	(b) What did the symptoms mean to you?	(i) Did the symptoms mean a serious disease or just some common disease that did not require immediate action?
	(c) What did you do when you recognized these symptoms?	(i) Did you talk to somebody about your symptoms?
		(ii) Who were these others you consulted?
		(iii) What were you told about the symptoms in terms of causes, consequences and management?
**2. Considered consequences of the disease; Illness stage**	(a) What made you feel that you needed to seek care out of your home?	(i) Where did you first go to seek care with these symptoms?
		(ii) Why did you go there?
		(iii) Where else did you go for treatment?
	(b) How was the decision made?	(i) Who were involved in the decision making process on seeking care?
		(ii) What were considered? – including severity, availability of money, previous experience with the disease.
**3. Behavioural stage**	(a) From the time you recognized that the symptoms were serious enough and needed visiting a health worker	(i) How long did you take to go to the hospital?
		(ii) Why did you take the time you took?
	(b) Were you referred to this hospital? How long did it take you to come to this hospital from that time of referral?	
	(c) Why did it take you that time?	
**4. Diagnostic stage**	(a) When you came to this hospital, how long did it take to have the result of the test for cancer back?	
	(b) Why did it take the time it took for you to receive the result?	
**5. Curability; Treatment stage**	(a) After receiving your cancer result, how long did it take you to receive the needed treatment?	(i) Tell us the time in days or weeks or months that passed from time you got cancer result to time when you got treatment you think was for treating the cancer itself.
	(b) Do you think that this kind of cancer can get cured?	(i) If you think it can get cured (or cannot cure), why do you think so?

### Interviews

Fourteen interviews were conducted by ADM with the help of two trained research assistants. At each interview, there were two people – the interviewer and an assistant who made field notes and operated a digital audio-recorder. The research assistants are university graduates with experiences in social sciences research and HIV patient counselling. Each research assistant also conducted two interviews; both had also participated in the translation and pretesting of the interview guide. Interviews lasted between 45 and 90 minutes. During the interviews participants’ recall of significant dates such as onset of symptoms and timing of their decision to seek healthcare was helped by the use of significant landmark events [21] including Christmas, Easter, independence day, onset of the civil war and encampment, the date of other co-morbidities such as HIV diagnosis and start of antiretroviral therapy, dates of births of children or first born, and dates of presidential elections.

All interviews began with an open-ended question, “Please tell me about the illness you have which is now known to be cervical cancer”; followed by prompt questions, “What were the first symptoms, when did the first symptoms start, what did you think was causing the problem and what have you done about the symptoms since onset up to the time you came to this hospital?” Participants were allowed to discuss their experiences without interference. The interview guide was used to facilitate the discussions for all patients. Detailed field notes and audio recordings were used to ensure capture of both verbal and non-verbal aspects of the interviews. All interviews were undertaken in a quiet room within the hospital premises. Interviews were completed within three months of the diagnosis of cervical cancer, and the majority of participants were interviewed in Luo/Acholi. Participants’ cross-checking and repeat interviews were not done. However, during the interviews, responses provided were summarized and read back to participants who confirmed or amended responses at the end of each section in the guide.

Interviews continued until the 18th patient when concurrent analysis revealed that data saturation had been reached and there were no new themes emerging [22].

### Data analysis

Transcription and analysis was an iterative process, starting after the first four interviews and continuing throughout data collection. Field notes and recorded interviews were transcribed and translated verbatim into English by the male research assistant who was present at 16 of the 18 interviews. All transcripts were reviewed against audio recordings by ADM (conversant with local language and English) to ensure accuracy in data capture, transcription and translation. The English versions of the transcripts were then read several times by ADM and ESO to gain familiarity with the data before they independently developed codes. A codebook for analysis was developed through consensus in a meeting between ADM and ESO. Data were analyzed using thematic analysis technique [23,24] with the aid of ATLAS.ti v6.1 software. Sub-themes and themes were agreed on through consensus in a series of meetings between the authors. ADM and ESO shared and revised the subthemes and themes with the research assistants in a one-day meeting to ensure that themes contained all the important issues expressed during interviews. Further data collection and analysis were informed by the themes from earlier interviews.

During data analysis and reporting we used the Model of Pathways to Treatment to provide the theoretical underpinning for the different intervals in the help-seeking trajectory [16,17]. We focused on the first two parts of the model; the appraisal interval which starts from the onset of symptoms to perceiving that one is ill and needs to visit a healthcare professional, and the help-seeking interval which starts from the decision to seek care to eventually visiting the healthcare professional. Total time to diagnosis comprise the time from appraisal through help-seeking to diagnosis of cancer. Intervals were calculated in months based on patient’s recall. Unfortunately exact dates of first visits to the peripheral primary care health professionals were difficult to recall accurately by patients and we could also not independently confirm, therefore total time to diagnosis rather than presentation is reported [25]. Mid-months and mid-years were used as dates of events when necessary.

Throughout the Results section, representative verbatim quotes are referred to by the patient’s study number (1 – 18), total time to diagnosis in months and FIGO cancer stage.

### Ethical approval

Makerere University School of Medicine Research & Ethics Committee (SOMREC) and the Uganda National Council of Science and Technology (UNCST) provided approval for this study. Further institutional approvals were provided by the institutional review committees (IRCs) of Lacor and Gulu hospitals. Written individual informed consents were obtained from participants before interviews. Verbal consents to audio record interviews were also obtained from each participant. We provided participants with modest amount of money not exceeding 5.0 USD in lieu of refreshments following interviews.

## Results

### Study respondents

The median age of the 18 participants was 42 years (range 35–56) with a range in parity from 0–11. Most participants presented with advanced stage disease (Table 2). Regarding occupation, most participants were subsistent farmers occupied with cultivating land and caring for their families. Two participants were involved in small scale businesses while one was formally employed as office secretary. Thirteen were married, three widowed and two separated. The median time interval from first noticing a symptom to diagnosis at the study hospitals was 20 months (range 2–140 months) (Table 3).

**Table 2:** **Socio-demographic characteristics and symptoms of cervical cancer**

**Participant**	**Age at diagnosis (years)**	**First three symptoms of illness in order of occurrence**					**FIGO stage**
		**Inter-menstrual bleeding**	**Post-coital bleeding**	**Post-menopausal bleeding**	**Lower abdominal pain**	**Vaginal discharge (foul smelling, pus-like, watery)**	
P1	35–39		1st		3rd	2nd	Missing
P2	35–39	1st				2nd	1B
P3	40–44						4A
P4	45–49	1st			2nd		1B
P5	40–44	2nd			3rd	1st	2B
P6	55–59			1st	3rd	2nd	3B
P7	40–44				1st	2nd	1A
P8	40–44	1st			2nd		1B
P9	45–49	1st			3rd	2nd	2A
P10	40–44	2nd			3rd	1st	3B
P11	40–44	2nd			1st		1A
P12	35–39	2nd	1st			3rd	2A
P13	55–59			3rd	1st	2nd	3A
P14	35–39		3rd		1st	2nd	2B
P15	35–39		3rd		2nd	1st	3B
P16	45–49	1st			2nd		3B
P17	50–54			1st	3rd	2nd	3B
P18	35–39	1st			3rd	2nd	2A

FIGO: International federation of Gynaecology and Obstetrics.

**Table 3:** **Patients’ first attributions of symptoms and total time to diagnosis (TTD)**

**Participant**	**TTD (Months)**	**FIGO stage**	**First attributions of illness symptoms**								
			**Retained condom**	**Intestinal worms**	**Genital infections (syphilis, gonorrhoea and Candida)**	**Menstrual problems (Menopause and other menstrual abnormalities)**	**Uterine swelling**	**No clue on cause**	**HIV and/or its complications**	**Cervical cancer**	**Other serious illness**
P10	2	3B						X			
P11	2	1A				X					
P7	3	1A	X								
P8	3	1B				X					
P4	4	1B								X	
P3	7	4A			X						
P15	10	3B			X						
P18	10	2A									X
P9	19	2A				X					
P16	21	3B			X						
P13	24	3A					X				
P12	26	2A							X		
P2	30	1B			X						
P5	39	2B							X		
P6	72	3B				X					
P14	72	2B			X						
P17	72	3B		X						x	
P1	Missing	140			X						

### Appraisal interval

We identified an iterative process during the appraisal interval, which involved initial symptom recognition, attribution of symptom/s, lay consultations with family and friends and self-management, and perceived personal risk of cervical cancer.

#### Initial symptom recognition

Most participants reported abnormal vaginal bleeding as their initial symptom, usually painless and unexpected. The vaginal bleeding started and progressed in varying patterns; some respondents experienced painless vaginal bleeding only after sexual activity, while others had unprovoked, episodes of inter-menstrual bleeding.

*“It first started towards the end of the year 2000. I could bleed after meeting a man in bed. I used not to have the pain. When I did not meet the man . . . no blood would come”,****(P1, 140months, missing stage)****.*

Six participants reported painless vaginal bleeding starting at about menopause onset or several years post-menopause. Peri-menopausal bleeding was often not perceived as a sign of serious illness but rather as the expected onset of menopause or restart of menstruation.

*“It started five years after I had stopped seeing my periods; I started bleeding like menstruation. The bleeding was fluctuating and could last between four to five days. I thought it would go away but it persisted and then I started telling other people that I had stopped this thing but it is still coming”,****(P6, 72 months, 3B)****.*

Unexplained excessive vaginal discharge, often itchy and/or foul smelling, was another common presenting symptom:

*“The first symptom was waist pain and vaginal milk-like discharge that was causing me a lot of itching in the genitals”,****(P2, 30 months, 1B)****.*

#### Attribution of initial symptom/s

Most participants attributed the initial symptoms of illnesses to sexually transmitted diseases (STD) e.g. syphilis and/or gonorrhoea, contracted from their husbands who were often suspected of being involved in sexual relationships with other women.

*“I thought it was syphilis. Whenever we had sex blood would flow. I thought it would stop, but over six months the bleeding was just increasing in amount and so I then came to this hospital”,****(P17, 72 months, 3B)****.*

*“Worries started when bleeding started to come with clots. The first thing that came to my mind was that my husband had given me syphilis. I went for injections for syphilis, which also helped for some time to relieve the pain”,****(P2, 30 months, 1B)****.*

Only a few participants who all reported they had heard about symptoms of cervical cancer, immediately attributed their symptoms as possible cancer.

*“People were talking about a disease that affects the uterus. I had already got some information from the radio sensitization program and immediately I saw these symptoms, I straight away concluded it was cervical cancer”,****(P4, 4 months, 1B)****.*

Symptom attribution was also influenced by their healthcare professionals’ diagnoses or advice. Participants who visited primary healthcare professionals during the early phase of their illnesses and obtained either no or a benign diagnosis hoped the symptoms would subside with treatment provided by the healthcare professionals at some point.

*“I started getting treatment from the clinics two years ago (since 2011) when pain started. I have been going there for a long time and I have bought a lot of medicine where they (primary healthcare professionals) tell me this one will now cure you but the day I stop taking the medicine the pain would even be more serious”,****(P13, 24 months, 3A)****.*

Participants who were told by primary healthcare professionals that their symptoms could be due to cancer reported seeking care promptly in the study hospitals.

*“It was because of the advice from that medical doctor . . . The doctor who did the ultrasound is the one who told me to rush to this hospital immediately because of the results of the examination that he had done. He was suspecting cancer but he could not confirm”,****(P15, 10 months, 3B)****.*

About half the participants thought that their abnormal vaginal bleeding symptoms were a result of past or current use of hormonal oral contraceptives, implants or injection contraceptives; these medicines were variously blamed for causing cervical cancer, weight gain and obesity and infertility in women.

*“I also thought that because I used some birth control pills . . . I used this one where they insert in your arms; I also took some orally”,****(P12, 26 months, 2A).***

Other co-morbidities also influenced symptom attribution, while current or previous life circumstances seemed to shape their symptom interpretation. For instance, participants living with HIV/AIDS attributed any new symptoms to HIV and its complications.

*“I thought it was the different HIV signs that come in various forms . . . In May 2009, when I started getting the pain, I started thinking it was either cervical cancer or TB. I thought it was TB of the stomach”,****(P5, 39 months, 2B)****.*

In general, illness interpretation depended on a number of personal and illness characteristics including individual’s previous knowledge of how certain illnesses present, previous experiences with certain illnesses or commonality of the illness in the community (Table 3).

#### Lay consultations and self-management

Participants’ interpretations of symptoms and speed of progression of the illness influenced the steps undertaken to deal with the abnormal bodily experiences. The majority of participants sought some forms of help within the home including self-medications and varying degree of consultations with husbands, relatives and friends. These conversations influenced participants’ decisions and timing of medical help-seeking for the symptoms. Reinforcing advice often fostered immediate help-seeking. No instances of false reassurances leading to increased help-seeking interval were noted among these participants.

*“I first told my husband. My husband told me to go to the hospital immediately. Then there was a lady teacher nearby; she used to tell me that I could be suffering from cancer and I needed to go to hospital immediately”,****(P15, 10 months, 3B)****.*

For most people, lay consultations served many purposes including the search for confirming views on what participants thought of their illnesses, obtaining more information about what might be causing the symptoms and what to do, and also fostered solidarity.

*“I shared my problems with my husband; he told me to go for treatment in the hospital; I told him because I wanted him to support me and also to let him know because we are now one person”,****(P4, 4 months, 1B)****.*

In contrast, a substantial minority were guarded with their symptoms because of concerns that people may do nothing to help, or even mock them or make them become the focal point for discussions in the community because the illnesses affected a sacred/private part of their bodies. In addition, some felt the symptoms were within their control, and they therefore either self-medicated or demanded particular tests from private clinics.

*“I took long without talking about the disease with anybody until I went to the clinic where I shared with a health worker. I thought that even if I share it with other people they will do nothing about it”,****(P2, 30 months, 1B)****.*

#### Perceived personal risk of cervical cancer

Perceived personal risk of cervical cancer played a key role in the appraisal of peoples’ symptoms. Most participants did not attribute the causes of their symptoms to cervical cancer because they did not perceive themselves to have been at risk of cervical cancer; some did not make the attributions because cervical cancer was an uncommon illness in the region.

*“I had never thought of getting this disease because the disease was not even common among our people. I first saw the disease only in one woman from the health centre where I had gone for treatment”,****(P4, 4 months, 1B)****.*

Previous history of cervical screening also influenced people’s perception of personal risk to cervical cancer. The few participants who had been screened for cervical cancer and understood their negative test result were reassured and believed they were no longer at risk.

*“No I did not imagine I would get cervical cancer because I went and tested twice and there was nothing”,****(P7, 3 months, 1A)****.*

Only one participant thought that she was at increased risk of cervical cancer as she understood that HIV infections increased risk to many diseases including cervical cancer.

*“Yes, I thought I was at risk. In 2000, I was told that when you have HIV you become vulnerable to cervical cancer. But before that I never thought I would get it”,****(P5, 39 months, 2B)****.*

Feeling at increased risk was also related to a woman’s age, menopausal status, and past sexual activity and infections. Participants who perceived that cervical cancer is related to sexual intercourse thought that older women who no longer engaged in sexual intercourse were not at increased risk for cervical cancer.

*“I kept asking myself how I got the disease when I am already this old; how can this disease come to me and yet my age is now beyond this common sexually transmitted illnesses. Even people kept saying mama, how can this illness disturb you at this age when you are already such an old woman”,****(P10, 2 months, 3B)****.*

### Help-seeking interval

The help-seeking interval was influenced by a number of trigger and delay factors. Symptoms perceived as severe or life threatening often triggered prompt help-seeking, while mild intermittent symptoms, and those appraised as normal bodily changes related to age or the menopause, often led to delays in help-seeking. Some participants consulted with primary healthcare professionals several times before being referred or self-referring to hospital.

#### Triggers for early help-seeking

Prompt help-seeking was often triggered by life-threatening, persistent or worsening symptoms, the appearance of new symptoms, or a lack of response to self-treatments. Participants with alarming symptoms such as heavy vaginal bleeding and severe pain sought care in the higher level health facilities within a shorter time from symptom onset. However, even with these symptoms, the diagnosis of cancer was sometimes not made immediately by the healthcare professionals.

*“The first sign started on the 13th of April, it started with a lot of blood flow. It started when I went out to urinate . . . Luckily it was almost morning; so I then came to Gulu hospital straight”,****(P8, 3 months, 1B).***

Although this respondent (P8, 3 months, 1B) attended care immediately following first symptom recognition, the diagnosis of cervical cancer was not made at the initial visit to the hospital; she was diagnosed 3 months later in another hospital.

Most participants sought help when the amount of vaginal bleeding increased or became more regular and burdensome, or their vaginal discharge became foul smelling, and or intensely itchy.

*“Previously I would get the discharge but I could wear one panty from morning up to evening, but with this one the flow was too much. It was just the quantity which was too much and I decided to go to the clinic”,****(P2, 30 months, 1B).***

All participants promptly sought help outside their homes and or in medical facilities when pain became a dominant feature of their illnesses.

*“What made me to start seeking for medical attention was because of the pain and bleeding from the genital. It instilled in me big fear because sexually transmitted infections when it starts causing vaginal discharge it means it has caused damage already. That is why when it started it took me only three days! I had to go to the clinic to seek medical attention”,****(P5, 39 months, 2B)****.*

Most also sought care when symptoms including pain interfered with their daily activities.

*“The pain was so much that I could not even move up a hill; when the pain starts, I need to stop immediately. When it comes and starts pinching me, I need to stop or lie down - even when I am eating”,****(P13, 24 months, 3A)****.*

#### Factors associated with prolonged time to help-seeking

Most participants did not associate scanty vaginal bleeding with possible cervical cancer or any other serious disease until it persisted or increased in amounts. Understanding of a woman’s bodily processes seemed poor, especially among the older women who associated irregular abnormal painless vaginal bleeding with the onset of the menopause. They only sought help when another symptom such as pain became prominent.

*“I never took this blood seriously because people were saying that for older women like us who are about to see the end of our menstrual periods there are some small blood drops which comes on and off. After this blood flow I stayed for about six months then the blood flow now started coming regularly; after the blood flow I started feeling pain on and off in my lower abdomen. From that time I started thinking that this could be a serious illness, so I started asking people for ideas about the problem”,****(P9, 19 months, 2A).***

Most participants who perceived their symptoms as due to a serious illness attended care in nearest health units promptly. However a number of healthcare factors were associated with prolonging time to diagnosis. First, some people were treated for other conditions, often over a long time, and this may have led to delayed diagnoses and initiation of appropriate treatment.

*“The first symptom was waist pain and vaginal milk-like discharge. I first went to CX centre clinic in Gulu and I was given tablets. I later proceeded to MX clinic and I was examined and told my uterus is dirty . . . I was given tablets to take for two weeks. The bleeding and discharge stopped while I was on treatment. When I went back to continue with my business, the sickness intensified between the months of July and August of 2011 with vaginal discharge of water coupled with much bleeding . . . September 2011, I came back to MX clinic and I was told to go to Lacor hospital”,****(P2, 30 months, 1B)****.*

Second, even when the peripheral health centres and hospitals suspected cervical cancer or some other serious illnesses, some people experienced problems with competing responsibilities, while others were unable to immediately afford the cost of transport and care in the referral hospitals. This is manifest in the quotations that follow below.

*“Issues to do with money, and because there are small children at home and there are others in school; and there are things that should be left at home for feeding them and even me when I come here there would be no one to take care of me from here because my husband must remain home to take care of the children so I went and brought my mother and she is the one who is taking care of me from here”,****(P18, 10 months, 2A)****.*

*“It was in the year 2000 that I went for HIV test and was found to be positive. In August 2000 I conceived and started experiencing vagina bleeding for a period of six months. After that I delivered and stayed without any problem until 2009 when I started discharging watery substance that could cause serious itching . . . I went with it to the hospital, I was given treatment and I got better. In May 2010, I started experiencing pain . . . Later I started experiencing change in my menstrual cycle that I could not explain; bleeding could last between 10 -12 days. I went to JX hospital (1 kilometre from her home) and I was given tablets to take, but there was no change in my condition. This made me to go to IJX (360 kilometres away, in the capital city) on the 14th August 2010 and the doctor suspected my problem to be cancer. The doctor told me they can remove the uterus and cancer at a cost of UGX 1,800,000 ($720.0). I could not afford that amount and that made me to come back to look for another alternative. I went back to JX hospital, on 17th August 2012 . . . and referred me to Lacor hospital where I came. . . 24th August 2012 confirmed I have cervical cancer”,****(P5, 39 months, 2B)****.*

## Discussion

This paper reports findings from one of the first studies exploring symptom appraisal and help-seeking for symptomatic cervical cancer among patients in the low-income sub-Saharan country, Uganda. There is evidence that help-seeking for some women with cervical cancer symptoms started immediately following symptom recognition and appraisal. More women had a longer time to presentation depending on; the nature of initial symptoms (onset, severity and persistence); the individual’s interpretations of the initial symptom/s and advice from lay people and family members; their perceived personal risk of cervical cancer; the existence of co-morbidities including HIV; and false reassurance from the primary care healthcare professionals first encountered. Our findings are consistent with evidence from high-income countries that help-seeking for illnesses depend on many factors including the individual’s social and cultural context [15], and the health care system.

Abnormal vaginal bleeding (including post-menopausal and/or post-coital), vaginal discharge and lower abdominal pain were the most common first symptoms reported by participants. Although these symptoms are recognized symptoms of cervical cancer [26], they also occur very commonly with other conditions including pelvic inflammatory diseases (PID) [27] and sexually transmitted diseases [28]. Failure to recognize the seriousness of symptoms, or attributing symptoms to other more common conditions, has been shown to delay appropriate help-seeking for symptoms of other cancers in the UK [29,30]. Furthermore, prompt recognition of potential cancer symptoms is associated with earlier diagnosis of breast, colorectal and lung cancers in the UK [31].

The majority of participants attributed initial symptoms of vaginal discharge and itching to STDs including syphilis and/or gonorrhoea. The availability of plausible alternative explanations for symptoms prolonged the participants’ appraisal and help-seeking intervals. Furthermore, some participants had delayed diagnoses due to long-term treatment of these STDs. As cervical cancer symptoms occur in the pelvic region, participants may have attributed their symptoms as STDs; even more, they may have assumed that cervical cancer could be treated with the same regimens they have used for self-management of STDs. Once symptoms are interpreted as STDs, delay in help-seeking may be even longer because STDs have generally been stigmatized in most communities including Uganda [32,33]. Again, this suggests the importance of cancer symptom awareness campaigns and interventions to reduce stigma and discriminations associated with illnesses.

Cervical cancer awareness campaigns also need to promote prompt healthcare seeking among women with any gynaecological symptoms to allow early detection of cervical cancer. When STDs are common, they may occur concurrently with cervical cancer and patients may not be aware of subtle symptoms changes. Some participants had long duration of symptoms, for example, participant P1 reported symptoms over 140 months. She was HIV positive and a widow. While she attributed all her symptoms to cervical cancer, it is possible that these symptoms were due to other genital infections such as Chlamydia that are common among HIV positive women [34].

Participants who perceived their initial symptoms as not due to a serious disease, or whose symptoms were painless, intermittent or mild, were likely to take a longer time to seek professional care. These findings are similar to those found by Emery and colleagues who interviewed rural Western Australian patients soon after diagnosis with breast, lung, colorectal and prostate cancers [10], and in the UK, where non-recognition of the seriousness of symptoms was the main patient-mediated factor that increased time to presentation for most cancers [29]. Other factors we identified as influencing the help-seeking duration were the distance to the nearest hospital, lack of money for travels and care, presence of co-morbidities such as HIV/AIDS, and perceived personal risk to cervical cancer. Most participants who did not perceive personal risk for cervical cancer minimized their cervical cancer symptoms, delayed seeking healthcare, and decided their symptoms were due to common benign diagnoses. This is consistent with results from interview studies with people recently diagnosed with cancer in high-income countries such as rural Australia [10], and the Netherlands [35]. It is important to develop cancer symptom awareness campaigns that include risk factors for cervical cancer and the people at increased risk so that women can correctly attribute their own symptoms and appropriately seek healthcare.

We found that consultations with lay people and family members usually shortened time to professional help-seeking. Similarly, rural women who discussed their breast symptoms with lay colleagues presented within a shorter time interval in rural Australia [10]. In the UK, it was reported that sanctioning of initial interpretations by significant others fostered immediate help-seeking while keeping silent about symptoms, either because of low expectations of inputs from lay colleagues, or fear of people talking about their symptoms, delayed professional health seeking [29]. Targeted health messages should aim to reduce perceived self-stigma associated with cervical cancer symptoms and STDs so that women may freely discuss their symptoms with family members and friends, and get encouragement by peers to seek appropriate care promptly.

The symptoms that triggered prompt help-seeking were severe abdominal pain, heavy vaginal bleeding, and/or excessive foul smelling vaginal discharges. These symptoms probably cause reasonable perceived threat to life and thus compel patients to seek healthcare as articulated in the health belief model [36]. The one participant who attributed her initial symptoms to cervical cancer also sought professional healthcare promptly. However, in a substantial minority of participants, women had prolonged diagnostic journeys despite recognizing the seriousness of their symptoms and seeking help promptly. An example was P5 (39 months, 2B) who sought care at an established health facility within one week of noting her first symptom but took more than 3 years to receive her diagnosis. Such a long diagnostic interval raises concern about the need to augment healthcare professional training around the recognition of common cancer symptoms including those for cervical cancer.

### Strengths and limitations

The main strength of this study is the use of validated theoretical models to underpin the data collection - the General Model of Total Patient Delay- and data analysis - the Model of Pathways to Treatment. The use of semi-structured interviews enabled patients to speak freely about their illness experiences from onset to diagnosis. Conducting interviews within three months of diagnosis is likely to have minimized recall bias.

The study is limited by use of retrospective recall to determine the exact dates of symptoms onset and decision to seek medical help. It was often difficult for the participants to accurately report the timing of their symptoms, or whether symptoms were actually due to cervical cancer. Misattribution of many different earlier symptoms may occur when the symptoms are common and shared by many diseases, and/or when the symptoms are chronic and long-standing [25]. However, in a qualitative study such as this, the inability to accurately determine the length of the appraisal, help-seeking and diagnostic intervals does not affect the value of patient perceptions about the help-seeking journey. Other potential sources of bias included the male gender of ADM and one of the research assistants, participation bias by more motivated patients and interpretations based on previous experiences. The female research assistant was involved to temper the effect of male interviewers while interpretative bias was minimized by discussion of themes by the authors who have different training, experiences and research backgrounds.

### Clinical and research implications

Our findings have important implications for care, research and policy. Regarding improvement in care, it is crucial to encourage women to seek professional help once self-help has failed to relieve symptoms. It is concerning that vaginal bleeding starting more than one year after previous menstrual bleeding were trivialized; rather than recognizing as a symptom of disease, post-menopausal bleeding was considered either as a restart of menstruation or something not serious. This may suggest a lack of awareness of menstrual cycle, meaning of menopause, and symptoms of cervical cancer. Interventions to create and /or increase community awareness about menstruation, normal and abnormal menopausal changes, and symptoms of cervical cancer may serve to avert prolonged delays to diagnosis resulting from alternative benign explanations or trivializations of symptoms of cancer. In the UK, general practitioners recognized the need to increase women’s awareness of gynaecological symptoms as a way of promoting prompt help-seeking [37]. There is also evidence from LMICs that creating public awareness about cancer improves early diagnosis of cervical cancer with women presenting with more early stage disease in the intervention areas [38-40].

The Ugandan healthcare system comprises several layers of expert training but it appears that some healthcare professionals at the lower level of training may also have clinical challenges in detecting symptoms and signs of cervical cancer, and referring appropriately for definitive diagnosis. In this study most participants visited the lower level healthcare facilities several times before eventually getting referral to the study hospitals. Similar findings of healthcare professionals accounting for delay in diagnosis and/or referral were reported in a South Africa study [12]. Both studies support the call for appropriate training of these lower level healthcare professionals to identify potential symptoms and signs of cervical cancer, acquire clinical techniques to diagnose cervical cancer, and/or promptly refer patients with symptoms suggestive of cervical cancer to centres where diagnostic capabilities exist.

Interventions such as further education for healthcare professionals have been shown to contribute to down-staging of cancers and are affordable especially where population based screening are not available [41]. Further researches to assess the clinical and cost effectiveness of down-staging through enhanced education is needed in the sub Saharan African countries where there is high incidence of cervical cancer. If found applicable, then it could be important for policymakers in the LMICs to further adopt such measures to increase the proportion of invasive cancers diagnosed at early stages.

With regards to further evidence needed from research, quantitative studies to accurately determine appraisal, help-seeking and diagnostic intervals using validated models, and to compare the respective intervals with cancer stage at diagnosis, is needed in the LMICs. These would provide evidence for policymakers on promoting awareness for symptoms of cervical and other cancers. These would also provide further evidence to underpin programmed further training and education for healthcare professionals, particularly in the lower level healthcare facilities in Uganda.

## Conclusions

Cervical cancer patients present with symptoms that are frequently attributed to normal bodily changes or common illnesses including sexually transmitted diseases. Lay consultations with family and community members tend to encourage self-management and early help-seeking, while not disclosing symptoms may delay help-seeking. Awareness campaigns to increase awareness of cervical cancer symptoms at population level, and educational programs for healthcare professionals, may help to improve early symptom recognition and timely help-seeking and diagnosis among people with symptoms suspicious of cervical cancer.

Our study is one of the first such studies from a LMIC, and contributes evidence to the key factors that influence symptoms recognition, interpretation and appraisal, and professional help-seeking for symptoms of cervical cancer in a low-income country.
